# Long COVID: what is known and what gaps need to be addressed

**DOI:** 10.1093/bmb/ldad016

**Published:** 2023-07-11

**Authors:** Benjamin Krishna, Mark Wills, Nyaradzai Sithole

**Affiliations:** Cambridge Institute of Therapeutic Immunology & Infectious Disease (CITIID), Cambridge CB2 0AW, UK; Department of Medicine, University of Cambridge, Cambridge CB2 0QQ, UK; Cambridge Institute of Therapeutic Immunology & Infectious Disease (CITIID), Cambridge CB2 0AW, UK; Department of Medicine, University of Cambridge, Cambridge CB2 0QQ, UK; Cambridge Institute of Therapeutic Immunology & Infectious Disease (CITIID), Cambridge CB2 0AW, UK; Department of Medicine, University of Cambridge, Cambridge CB2 0QQ, UK; Department of Infectious Diseases, Cambridge University Hospitals NHS Foundation Trust, Cambridge CB2 0QQ, UK

**Keywords:** Long COVID, PASC, symptoms, causes, treatments

## Abstract

**Introduction:**

Long COVID is a chronic condition that follows after acute COVID-19 and is characterized by a wide range of persistent, cyclic symptoms.

**Sources of data:**

PubMed search for publications featuring ‘Long COVID’ or ‘post-acute sequelae of COVID-19’.

**Areas of agreement:**

Long COVID occurs frequently post-acute COVID-19, with a majority of people experiencing at least one symptom (such as cough, fatigue, myalgia, anosmia and dyspnoea) 4 weeks after infection.

**Areas of controversy:**

The specific symptoms and the minimum duration of symptoms required to be defined as Long COVID.

**Growing points:**

There is a consistent reduction in Long COVID incidence amongst vaccinated individuals, although the extent of this effect remains unclear.

**Areas timely for developing research:**

There is an urgent need to understand the causes of Long COVID, especially extreme fatigue more than 6 months after infection. We must understand who is at risk and whether reinfections similarly risk Long COVID.

## Introduction

Three years into the global COVID-19 pandemic, evidence from millions of infections shows that COVID-19 symptoms persist, and in some cases new symptoms emerge, in a significant proportion of individuals after the acute phase of infection. This syndrome is known as post-acute sequelae of COVID-19 (PASC) or Long COVID.^1^^,^^2^ The heterogeneity of Long COVID symptoms point towards this being a multi-syndromic and multi-phasic condition rather than a singular disease phenomenon. Patients report a plethora of relapsing and remitting symptoms for several weeks and months with some failing to recover after 2 years. The symptoms are broad in nature, reveal multi-system involvement and detrimentally affect patients’ quality of life.^1^^,^^3^ Although emerging data suggest that a proportion of symptoms resolve over time, many are nevertheless afflicted with life-altering symptoms more than 6 months after infection.^3^ Therefore, there is a pressing clinical need to identify the risk factors, and determine and understand the causes in order to facilitate development of much-needed treatments for Long COVID.

Published work on Long COVID suggests that a significant proportion of those with COVID-19 experience at least one ongoing symptom more than 4 weeks post-infection.^4–13^ As evidence emerges to show that in a significant proportion of patients, recovery from COVID-19 is slow, NICE guidelines have defined acute COVID-19 as lasting up to 4 weeks, ongoing symptomatic COVID as lasting 4–12 weeks and post-COVID-19 syndrome lasting more than 12 weeks.^14^ The NICE definition is appropriate as it helps to distinguish individuals experiencing a slow recovery from those with symptoms more classically associated with Long COVID. However, as data emerge and time passes, it is clear that there is a need for an objectively determined distinct group for those experiencing severe fatigue more than 6 months after infection, which is a particularly detrimental symptom and should be classified to reflect this.

The major issues that need to be resolved include accurate assessment of the prevalence of Long COVID, based on a universally accepted criteria of what symptoms constitute Long COVID after an accepted cut off time; identifying risk factors predictive of developing Long COVID; and understanding the molecular and pathophysiological basis behind Long COVID so that novel therapeutics can ultimately be designed to combat this life-distressing condition. This review aims to assimilate what is known so far on each of the aforementioned, major questions pertaining to Long COVID and should be used in addition to systemic reviews that have been published recently.^15–17^

## The prevalence of Long COVID is difficult to assess without stricter definitions of symptoms and symptom duration

It is important to quantify the prevalence of Long COVID in order to fully appreciate and understand the burden it bears on various facets of society. Studies estimate that anywhere between 0.2 and 80% of patients have at least one ongoing COVID-19 symptom from 2 months after infection.^13^^,^^18^^,^^19^ This extremely wide range of estimates is caused by several methodological differences in quantifying Long COVID as well as a lack of inclusion and exclusion criteria. First, studies that only include hospitalized patients tend to have higher estimates for ongoing symptoms compared with those that include non-critical patients.^8^^,^^20^^,^^21^ This is likely because of a correlation between severe COVID symptoms and the length of ongoing symptoms.^4^^,^^6^^,^^8^^,^^11^^,^^18^^,^^22–32^ Second, studies may test for subclinical changes of which the patients are unaware such as pulmonary and renal function.^8^ Third, recruitment practices also affect estimates; for example, studies that use population-level electronic health records (EHRs) may be less biassed than those that recruit through online surveys but EHRs will only detect patients with official diagnoses. Finally, the choice of, and duration of, symptoms for inclusion and exclusion criteria for diagnosis of Long COVID varies between different studies, resulting in significantly different prevalence estimates.

One major drawback to quantifying Long COVID is that many of the associated symptoms overlap with those commonly found in various other disease states. This is highlighted in studies that included a negative control containing people not believed to have had COVID-19. The Office for National Statistics found that although 5.0% reported Long COVID symptoms 12–16 weeks after infection, 3.4% of a control group who had not tested positive for COVID-19 did too.^33^ A study that looked at persistent symptoms 3 months post-infection in adolescents^34^ found that 16.2% of negative controls had three or more Long COVID symptoms, whereas 18% of negative controls in a study from Israel reported fatigue.^35^ Although some negative controls may have had COVID-19 (those with suspected COVID-19 are just as likely as those with confirmed COVID to have ongoing symptoms^3^) these reports raise the point that Long COVID may continue to be difficult to diagnose based on symptoms alone unless objective, molecular assays can be developed or specific symptoms defined. Until then, assessing the severity of symptoms, such as asking whether those experiencing fatigue are able to work and exercise, may help refine estimates and diagnoses of Long COVID. One such cautionary example comes from Garrigues et al.^24^ who show that despite over half of hospitalized patients experiencing ongoing symptoms 110 days after discharge, 69.1% of them were back at work and 71.8% had resumed physical activity. This highlights how patient-reported symptoms need to be scaled for their severity in order to understand the impact of Long COVID on their lives. As discussed later, there is now evidence that immunity to severe acute respiratory syndrome (SARS)-CoV-2, through either past infections or vaccination, reduces the risk of Long COVID. This means that not only is the risk of Long COVID hard to define but it likely changes over time.

Overall, these studies suggest that any single percentage value used to state the risk of Long COVID after COVID-19 must be taken with caution. Perhaps it would be best to discuss Long COVID risk in terms of specific symptom clusters at specific time points. Importantly, these observations suggest that Long COVID is not synonymous with a functional neurological disorder. Studies consistently find that those with confirmed COVID-19 have increased rates of various symptoms, and this effect appears to be stronger than other post-viral illnesses such as post-influenza.^36^

## Long COVID symptoms

The most common symptoms that persist for the first 6 months post-infection tend to be anosmia, chest pain, coughing, dyspnoea, fatigue and post-exertional malaise (PEM),^2–6^ with people commonly describing relapsing and remitting symptoms over time.^37^ Over 200 symptoms have been listed^15^ revealing multi-organ involvement including skin, kidneys, GI tract, endocrine, pulmonary, reproductive and cardiovascular systems as well as neuropsychiatric sequalae.^1^

Although the severity of symptoms can be hard to quantify because of the subjective nature of patient reports, severity of acute disease correlates with severity of Long COVID symptoms, and factors that worsen acute disease severity such as age and comorbidities increase Long COVID severity.^4^^,^^6^^,^^8^^,^^11^^,^^18^^,^^22–32^ Despite this, studies that have looked at relatively longer time points (6 months post-infection) still reveal Long COVID in those with relatively mild disease.^38^

Many of the early symptoms of Long COVID are likely because of recovery from viral immunopathology.^39^ Furthermore, those hospitalized with COVID-19 have high rates of readmission, all-cause mortality and respiratory, cardiovascular disease and diabetes, with higher rates amongst older people^40^^.^^41^ Indeed, higher viral loads seen in the severely ill would lead to more pathologies and a longer recovery.^42^ Histological data in mice suggest that Long COVID includes increased scarring of peripheral organs compared with influenza A virus infections, which could be because of increased SARS-CoV-2 RNA triggering a higher immune response.^43^ The study also noted increased interferon (IFN)-γ responses in the nervous system up to a month post-infection, particularly in the striatum, which is associated with chronic pain. Thalamic dysregulation might explain chronic pain, headache, myalgias, seizures, sleep and affective disorders, serving as a possible underlying factor in early Long COVID symptoms.^43^ Damage to the lung is expected and 4 months after discharge 50% of patients who were hospitalized with COVID-19 still had reduced lung functions below 80% of expected value as measured by diffusing lung capacity for carbon monoxide.^22^ Although patients on mechanical ventilation shed virus for much longer, a factor that may play a role in persistent symptoms,^44^ post-intensive care syndrome likely overlaps with many symptoms associated with Long COVID in those who required intensive care admission.^45–49^

The initial 6 months post-acute COVID-19 illness is characterized by a slow recovery for many patients.^9^ Taquet et al.^36^ also noticed that symptoms evolve over time from an early phase to a late phase around 6 months; some symptoms appear to occur together, whereas cognitive issues and myalgia do not, suggesting different origins for these symptom clusters.^36^ Given some symptoms only appear 90 days after infection,^36^ this suggests a case for either symptom relapses or evidence of new, intercurrent illnesses, such as a reinfection with SARS-CoV-2. Long COVID symptom trajectories over time possibly reveal clues pertaining to the underlying molecular mechanisms for these symptoms.

Although many recover from Long COVID within 6 months, some patients remain symptomatic and often show different or new onset symptom profiles compared with those experienced earlier after infection. Davis et al.^3^ identified symptom clusters that change over time for people suffering from Long COVID; symptoms such as fever, cough and sore throat tend to resolve early, whereas brain fog, bradycardia and myalgia occur later and persist for longer periods of time. Similar observations have been made elsewhere,^50^ including using an online survey, that noted symptoms associated with acute respiratory infections tend to resolve, whereas neuropsychiatric conditions emerge and persist^11^ (though as recruitment was through online support groups the participants surveyed likely reflect the most enthusiastic and therefore symptomatic respondents^37^). Our observations noted that in some patients, the neuropsychiatric symptoms resolved over time.^51^ Perhaps this evolution of symptoms over time points towards underlying mechanisms that cause Long COVID—ongoing symptomatic COVID-19 tends to mimic acute COVID-19 and suggests many people take months to recover from pathologies associated with a respiratory virus. Later symptoms are more consistent with post-viral fatigue. It is also interesting to note that fatigue tends to be associated with onset of disease, whereas brain fog and cognitive issues tend to emerge weeks after infection,^37^ and potentially more so in younger adults.^52^ Understanding the molecular causes of both symptom sets will help researchers develop treatments in the future and triage patients correctly for different treatments.

There are other, less common symptoms that include new allergies and anaphylaxis, which might be associated with Long COVID,^3^ and it would be interesting to understand how often these occur in an uninfected population before assigning these as new symptoms associated with Long COVID.

In addition to neurological conditions associated with Long COVID, cardiac damage caused by COVID-19 may not fully resolve, leading to increased long-term risk of cardiovascular disease.^53^^,^^54^ Screening will likely be required, especially for those hospitalized with COVID-19, for myocarditis and heart dysfunction as many may be at risk of myocardial infarction and other cardiovascular diseases.^55^ It is prudent to treat with anticoagulants in patients with imaging-confirmed venous thromboembolism.^53^^,^^56^^,^^57^ Therefore, the role of long-term treatment with anti-platelet and anti-coagulation therapy is under investigation to reduce the risk of cardiovascular disease.^1^

### Recovery from Long COVID

The rates of recovery from Long COVID are not well defined, either in terms of how quickly people recover or what percentage of people previously assigned a diagnosis of Long COVID have fully recovered to date. This is perhaps the most pertinent question for patient care.

Full symptom resolution over time likely resembles exponential decay, whereby many people recover quickly but there is a long tail end of people suffering for many months or even years. Consequently, exact definitions of Long COVID will affect calculations on recovery from Long COVID, as would definitions of recovery. It is plausible that a subpopulation of patients might return to work and resume regular exercise but still personally feel not back to their pre-morbid baseline functional status.

There are some studies measuring recovery from Long COVID that can provide clues towards a rate of recovery. One survey-based study saw 14.5, 5.1 and 2.2% of respondents reporting Long COVID symptoms at 4, 8 and 12 weeks post-infection.^9^ Comparably similar numbers were observed in Australia, with 4% of COVID-19 patients reporting ongoing symptoms at 120 days post-infection. Another found that around 70% of patients with Long COVID showed a reduction in autoantibodies 12 months post-infection, and 75% reported no further COVID-related symptoms.^58^ Another study suggests that 42% of people have only ‘partially’ recovered at 18 months post-infection, although symptoms reported are only marginally higher than uninfected control cohorts making this claim harder to interpret.^59^

## Vaccination appears to reduce the risk of Long COVID

Vaccination is currently the only COVID-19 intervention with reproducible efficacy against Long COVID. Research in Israel revealed reduced rates of Long COVID symptoms at 4 months post-infection in doubly vaccinated individuals.^35^ Here, vaccination reduced symptoms in infected individuals to such an extent that there was no observable difference in Long COVID symptoms between vaccinated individuals and uninfected individuals. Understandably, symptoms such as headaches, shortness of breath and persistent coughs will be reported in the population regardless of COVID-19 infection.^12^ Vaccination also reduced the risk of continuing symptoms at 28 days after infection in the USA^60^ and gave a 35% reduction at 12 weeks post-infection in the UK,^61^ a 79% reduction in Long COVID fatigue in one retrospective study^62^ and 15% reduction at 6 months post-infection in US veterans.^63^ The differences in magnitude of effect could be because of differences study populations (US veterans, for example, are 85% male and have a range of additional health problems), but also because of different thresholds for symptom severity and durations required to qualify for Long COVID.

It is unclear why vaccination might reduce the prevalence of Long COVID although one can hypothesize a number of plausible mechanisms. One might expect that vaccination reduces the number of SARS-CoV-2 infections that would reduce cases of Long COVID. However, as the number of SARS-CoV-2 infections has been higher since the emergence of the omicron variant^64^ with little to no increase in Long COVID cases,^65^ this is unlikely to be the whole answer. Vaccination also likely reduces Long COVID symptoms, to the point where some people’s symptoms are subclinical.

Reduction in Long COVID symptoms through vaccination could be achieved via a number of mechanisms, one of which is likely to be through reduction in severity of acute COVID-19 symptoms leading to reduced viral pathology that translates to fewer cases of Long COVID. For studies that measure chronic symptoms at 28 days and 12 weeks, it is plausible that vaccination results in reduction in viral load and virus-induced pathology, both of which translate to reduced Long COVID prevalence.^66^ Studies looking at 6 months post-infection (which tended to see lower effect sizes) may be observing a weaker effect of vaccination on post-viral fatigue, the most prevalent chronic symptom in Long COVID. This may also be caused by reduced early viral pathology, but understandably there are potentially other plausible mechanisms. Reduced viral load might reduce the risk of vasculopathy, for example. Other possibilities include reduced likelihood of persistent viral infections, and direction of the immune system away from autoimmunity. The interplay between pre-existing co-morbidities and acute disease severity must also be considered.

One important question that arises regarding vaccination is whether we will see a rise of Long COVID cases once vaccination booster programmes in the non-vulnerable population are stopped. Assuming that immunity will wane after final vaccination, some individuals will experience breakthrough infections and then Long COVID. If this occurs, it would suggest that a certain threshold of immunity is required to protect against Long COVID.

### Reinfections with SARS-CoV-2 likely show a reduced risk of Long COVID symptoms

The fact that vaccination appears to protect against Long COVID suggests that anti-SARS-CoV-2 immunity in general protects against Long COVID. This might suggest that second, third and further infections with SARS-CoV-2 would have reduced likelihood of Long COVID symptoms. As the majority of people have likely been infected multiple times with SARS-CoV-2, this question is now pertinent.^67^ Al-Aly et al.^63^ appear to find that each reinfection increases the likelihood of Long COVID, but each subsequent infection has a marginally lower risk. The cohort of patients from the Veterans Health Administration hospitals is older and has more co-morbidities than the general population, which may affect data analysis. Additionally, the authors do not include data from the acute phase of first infections, which may skew analysis between first and second infections. This work does, however, suggest that the risk of experiencing Long COVID symptoms from a reinfection with SARS-CoV-2 is not zero. Data from the National Office of Statistics agree that second infections also carry a risk of reported Long COVID symptoms; however, they suggest that the likelihood of these symptoms is reduced by 28%.^68^ As more longitudinal data emerge, and ideally with stricter definitions of Long COVID, we may be able to understand how damaging SARS-CoV-2 reinfections are for society.

### Potential causes of Long COVID

A deep understanding of Long COVID pathophysiology may help guide treatment options for patients.^69^ At least six potential mechanisms have been suggested to cause Long COVID: persistent/permanent organ damage caused by acute infection (which has been discussed above), persistent viral infection, autoimmunity, immune dysregulation, vascular disease/coagulopathy and reactivation of latent viruses.

Persistent SARS-CoV-2 infections in niches within the body could cause Long COVID by triggering persistent immune responses, which trigger symptoms such as fatigue, general malaise and myalgia. SARS-CoV-2 RNA has been detected in the brain and other body parts in COVID-19 autopsies (albeit without clear signs of inflammation, suggesting infection may be very low level).^70^ Elevated T cells in the lungs of people experiencing pulmonary PASC, persistent immune activation in patients with Long COVID and elevated spontaneous IFN gamma production have also been interpreted as indicating the presence of persistent virus.^71–73^ In one study, spike protein was found in 60% of patients with Long COVID at 12 months post-infection.^74^ Beyond this, there is evidence that virus may persist in the host post-acute infection, and viral shedding in stool 7 months after infection suggests that the virus may persist in the gut in cryptic sites of some people.^75^^,^^76^ Supporting this hypothesis, gastrointestinal-related Long COVID symptoms correlated with higher T-cell activation of new clonotypes, suggesting these cells emerge during the convalescence phase^66^ and it is possible that lingering virus in the gastrointestinal tract is responsible for the symptoms.^77^ Similar analyses have found SARS-CoV-2 nucleocapsid protein in breast tissue of one patient with Long COVID^78^; however, a larger study did not find persistent virus protein in the blood.^79^ Vaccination could therefore reduce Long COVID incidence by limiting the formation of cryptic viral reservoirs either via reduction of peak viraemia or by aiding in the clearance of virus from niche sites. Antiviral therapy could be used to clear out remaining virus or inhibit virus antigen production,^80^ but it would require a combination of different antivirals acting at different stages of the viral life cycle in order to minimize the chances of virulent resistant mutations evolving akin to what has been a successful strategy in treating Human Immunodeficiency Virus with combined highly active antiretrovirals.

Alternatively, Long COVID may be caused by immunity to self-antigens triggered by COVID-19. The possibility of autoimmunity induced by SARS-CoV-2 is not unthinkable as it is known that some cases of autoimmune diseases are either unmasked by viral illness or initiated following a viral illness.^81^ Vaccination prior to infection may help to direct the immune system to target the virus while discouraging immunity against self-antigens. Autoreactive T cells have been identified in severely sick patients and these may persist to cause Long COVID.^82^ Autoantibodies have also been found in patients with Long COVID,^58^^,^^66^ although these were present at diagnosis suggesting they were at subclinical levels before acute COVID-19.^66^ A number of studies have found a correlation between autoantibodies and severity of disease.^83–85^ It seems likely based on other studies that acute COVID induces these autoantibodies, especially in severely ill patients, and so the correlation with Long COVID may be a correlation with severe disease.

A third possibility is that Long COVID is caused by immune dysregulation. Histamine receptor antagonists have been used to treat Long COVID, and showed reduced symptom burden and T-cell perturbations, which the authors suggest may be playing a causal role in Long COVID.^38^ This is particularly interesting as it has been noted that some symptoms of Long COVID overlap with Mast Cell Activation Syndrome^3^^,^^86^ and multisystem inflammatory syndrome in children.^87^ Treatment with other anti-inflammatory agents such as anti-interleukin (IL)-6 have also been considered.^88^ Others suggest that C–C chemokine receptor type 5 expression is decreased in patients with Long COVID up to 8 weeks post-infection, leading to potential treatment of Long COVID with leronlimab,^89^ which warrants further investigation.

Reactivation of latent viruses, particularly the herpesviruses Epstein–Barr virus (EBV), human cytomegalovirus (HCMV) and human herpesvirus 6A and B have been seen in patients with Long COVID.^66^^,^^90–93^ Herpesvirus reactivations could potentially cause symptoms associated with Long COVID such as myalgia, malaise and fatigue, suggesting a possible mechanism for some Long COVID symptoms. It is also plausible that COVID-19 might trigger reactivation of latent herpesviruses, which are reactivated by stresses such as lymphopaenia and inflammation^94^; however, detection of herpesvirus reactivation might be coincidental with post-acute COVID-19 and not a causative agent of symptoms noted with Long COVID. Interestingly, it is possible that the reverse could be true: herpesvirus infections could induce ACE2 expression that exacerbates SARS-CoV-2 spread, as has been shown for HCMV,^95^ leading to increased immunopathology hence increased risk for Long COVID symptoms.

Microclots are deposits of fibrinogen that are resistant to fibrinolysis because of their amyloid structure^17^ and have been observed in Long COVID.^96^^,^^97^ These are hypothesized to form because of spike protein interaction with fibrinogen,^98^ which starts during acute COVID-19 but persists during Long COVID. Microclots occlude blood flow that, in turn, could lead to multi-organ involvement and are a tantalizing mechanism for symptoms such as myalgia and erectile dysfunction. Additionally, these microclots appear to entrap inflammatory mediators,^99^ which might lead to immune dysregulation and herpesvirus reactivation. There is some evidence that monocytes express pro-thrombotic genes after SARS-CoV-2 infection.^100^ This raises a number of questions, such as why only some patients go on to develop Long COVID, if microclots/prothrombotic states are prevalent in most acute COVID illness phases and why/how vaccination might help to alleviate this. Whether patients treated with anticoagulants experience fewer Long COVID symptoms would be interesting to see; however, the universal use of therapeutic dose anticoagulation in COVID-19 infection was discontinued as more data showed that the risk of bleeding outweighed generic use of anticoagulation.

Notably, these causes are not mutually exclusive and in some cases there is a lot of overlap. As an example, a longer period of SARS-CoV-2 infection (either persistent or not) could lead to more viral pathology, causing organ damage and inflammation that would cause stress that reactivates latent herpesviruses. Vasculopathy and autoimmunity similarly may cause organ damage, inflammation and viral reactivation. Consequently, viral persistence could increase the risk of dysregulated coagulation.

## Long COVID and post-viral syndrome

Post-viral fatigue is perhaps most associated with EBV but has also been documented after 1918 pandemic influenza, Influenza A(H1N1)^101^^,^^102^ and Ebolavirus epidemics as well as Tick-born encephalitis.^103^ Intriguingly, very high rates of post-viral fatigue appear after SARS-CoV-1 infections and Middle Eastern Respiratory Syndrome (MERS) as high as 50 and 40% suffering with chronic fatigue 1 and 4 years, respectively, post-infection. This may indicate that post-viral fatigue is especially prevalent after coronavirus infections.^104–110^ There is paucity of evidence for fatigue after seasonal coronavirus infections, though this could be because of their lack of clear symptom profile and/or research interest. Worryingly, despite post-viral fatigue being known for several decades, there are hardly any reliable treatment options available,^111^ hence treatments for Long COVID will be novel in nature and might go a long way in helping patients with post-viral/infectious fatigue.

Potential overlaps between Long COVID, postural orthostatic tachycardia syndrome, myalgic encephalomyelitis (ME) and/or chronic fatigue syndrome (CFS) have been investigated, based on the observation that these conditions have fatigue and myalgia as common symptoms.^3^ Indeed, up to 58% of people with Long COVID might meet the categorization of ME/CFS.^112^ Post-viral fatigue has been associated with polymorphisms in genes encoding IFN-γ, IL-10, IL-6 and tumour necrosis factor-α.^113^^,^^114^ Post-mononucleosis chronic fatigue is associated with changes in a range of inflammatory cytokines such as IL-6, IL-8 and IL-23.^115^ As dysregulated cytokine expression has been seen in patients with Long COVID,^116^ though not consistently,^79^ these conditions may have similar molecular causes.

### Predicting Long COVID

There would be multiple benefits to predicting Long COVID in patients either before or during acute infection: future treatments could be administered more quickly; patients could receive specific care and predictors of Long COVID might inform mechanistic studies. As EBV reactivation has been linked to Long COVID,^66^ detection of high viral loads may help direct treatments.^42^ Autoantibodies, as discussed above, correlate with Long COVID and may also be used in the future to predict Long COVID risk.^66^

It is unclear why more women report Long COVID symptoms.^37^^,^^79^^,^^117^ Although sampling bias is a tractable argument; nonetheless, Thompson et al.^13^ found more women with Long COVID diagnosis in the NHS EHRs, a measuring technique that might help to control for bias in survey reporting by requiring a clinician to agree with reported symptoms. Women were also more likely to have prolonged symptoms following SARS-CoV-1 infection, suggesting that there may be an underlying biological explanation associated with long-term symptoms following betacoronavirus infections and gender.^106^ Medical practitioners should expect, however, to see more women with Long COVID symptoms than men. Additionally, whether Long COVID affects sociologically disadvantaged and/or BAME patients, remains unclear.^1^ One study also identified a lack of rest during acute COVID-19 as a risk factor for developing Long COVID symptoms,^37^ and assessing this could play a role in reducing Long COVID or predicting risk in the future. Paxlovid (nirmatrelvir) reduces the risk of developing Long COVID in severely ill patients, indicating that to some extent, viral spread and replication plays a role in causing Long COVID.^117^ Therefore, it is plausible that those on antiviral therapies are less likely to develop Long COVID.

### Treatment trials for Long COVID

Treatments for Long COVID are currently in their infancy, mostly based on small studies and individual case reports.^15^ Exercise therapy has been proposed as a treatment for Long COVID; however, some worry that if exercise regimes are too strenuous or improperly supervised they could trigger PEM in some patients,^118^ as has been reported in other studies.^119^ Treatments focussing on alleviation of microclotting have shown that anticoagulants help to reduce Long COVID symptoms,^96^ as does apheresis.^120^ Apheresis may help through a range of mechanisms including reductions in markers of inflammation, autoantibodies and lipids.^121^

An aptamer, BC007, which blocks autoantibody binding to G protein-coupled receptors is being trialled,^122^ and its success provides circumstantial evidence to support the role of autoimmunity contributing to the pathogenesis of Long COVID symptoms. The reported success of histamine receptor antagonists already suggests this to be the case.^38^ If persistent virus replication is a cause of Long COVID then Paxlovid treatment might reduce viral replication and alleviate symptoms, as suggested in some cases.^117^^,^^123^

Overall, a range of approaches are being taken to treat Long COVID. Given that positive responses have been noted from a wide range of different treatments, this suggests that different approaches targeting different pathways may be required to alleviate the plethora of symptoms, that are likely because of different aetiologies.

## Conclusions

Long COVID describes a range of symptoms that contribute to the morbidity that occurs post-acute COVID-19. Now that the existence of Long COVID is well acknowledged, there is a need for increased awareness across the medical field and allied health professionals so that patients can be diagnosed and get targeted multi-disciplinary team management. A multi-disciplinary approach is vital given the multi-organ involvement in Long COVID.

Key to this is understanding the symptoms and duration of Long COVID. Prolonged symptoms 4 weeks after COVID-19 onset are extremely common, and initial symptoms are likely caused by virus-induced immunopathology, and/or post-intensive care syndromes. Severity of initial disease largely dictates the incidence of prolonged symptoms up to 6 months. After 6 months, symptom clusters appear to evolve and fatigue and brain fog dominate as symptoms, but the rates and severity of this condition are not yet fully characterized. There is a need to further define Long COVID into endotypes based on symptom groupings and characteristics that allow clearer dialogue and productive collaborations between researchers.

It seems unlikely right now that a single therapy will be effective for every patient under the broad category of Long COVID. Identifying treatments for patients is of the utmost priority but objectively classifying patients into subsets will significantly help to direct therapies.

Vaccination clearly reduces Long COVID rates, even though the magnitude of reduction has not yet been fully determined. An understanding of the molecular mechanism behind Long COVID would go a long way to help elucidate how vaccination reduces Long COVID prevalence and concomitantly assists in the development of treatments for Long COVID.

Although the high prevalence and magnitude of symptoms in Long COVID can partly be attributed to the fact that millions of people were infected in a very short space of time, hence the magnitude of the post-acute illness, Long COVID has been more noticeable than other post-infectious-related conditions following infection from different pathogens. There may be factors specific to SARS-CoV-2, such as molecular mimicry, which make chronic symptoms more likely. Given the high rates of persistent symptoms after SARS and MERS, this could be a feature of pandemic coronaviruses. If this is the case, what we learn about Long COVID in the next few years might help prepare strategies for other Long COVID-like diseases in the future.

## Author contributions

Benjamin Krishna (Writing—original draft, Writing—review & editing), Mark Wills (Funding acquisition, Writing—review & editing), and Nyaradzai Sithole (Funding acquisition, Writing—original draft, Writing—review & editing)

## Funding

This work was funded by a Wellcome award (225023/Z/22/Z) to BK and a National Institute for Health and Care Research award (G112259) to NS.

## Conflict of interest statement

The authors have no potential conflicts of interest.

## Data availability

No new data were generated or analyzed in support of this review.
